# Filler Influence on H_2_ Permeation Properties in Sulfur-CrossLinked Ethylene Propylene Diene Monomer Polymers Blended with Different Concentrations of Carbon Black and Silica Fillers

**DOI:** 10.3390/polym14030592

**Published:** 2022-02-01

**Authors:** Jae Kap Jung, Chang Hoon Lee, Un Bong Baek, Myung Chan Choi, Jong Woo Bae

**Affiliations:** 1 Hydrogen Energy Materials Research Center, Korea Research Institute of Standards and Science, Daejeon 34113, Korea; ubbaek@kriss.re.kr; 2 Department of Biochemical and Polymer Engineering, Chosun University, Gwangju 61452, Korea; artphys63@gmail.com; 3 Rubber Research Division, Korea Institute of Footwear & Leather Technology, Busan 47154, Korea; mcchoi@kiflt.re.kr (M.C.C.); jwbae@kiflt.re.kr (J.W.B.)

**Keywords:** ethylene propylene diene monomer, carbon black filler, silica filler, volumetric analysis, hydrogen uptake, diffusion, permeability

## Abstract

Filler effects on H_2_ permeation properties in sulfur-crosslinked ethylene propylene diene monomer (EPDM) polymers blended with two kinds of carbon black (CB) and silica fillers at different contents of 20 phr–60 phr are investigated by employing volumetric analysis in the pressure exposure range of 1.2 MPa~9.0 MPa. A linear relationship is observed between the sorbed amount and pressure for H_2_ gas, which is indicative of Henry’s law behavior. The hydrogen solubility of EPDM composites increases linearly with increasing filler content. The magnitude of hydrogen solubility for the filled EPDM composites is dependent on the filler type. The hydrogen solubility is divided into two contributions: hydrogen absorption in the EPDM polymer and hydrogen adsorption at the filler surface. Neat EPDM reveals pressure-dependent bulk diffusion behavior. However, with increasing filler content, the diffusivity for the filled EPDM composites is found to be independent of pressure. The magnitude of filler effects on the hydrogen permeation parameter is measured in the order of high abrasion furnace CB~semireinforcing furnace CB ˃ silica, whose effect is related to the specific surface area of CB particles and interfacial structure. The correlation between the permeation parameters and filler content (or crosslink density) is discussed.

## 1. Introduction

Ethylene propylene diene monomer (EPDM) [1,2,3] is a type of synthetic rubber made from ethylene, propylene, and a diene comonomer that enables crosslinking via sulfur vulcanization. EPDM is always used compounded with fillers, such as carbon black (CB) and silica, and with plasticizers, such as paraffinic oils. This material has useful rubbery properties only when crosslinked. Crosslinking mostly takes place via vulcanization with sulfur but is also accomplished with peroxides for improved heat resistance.

EPDM elastomers have been proven to be useful gas barrier materials in the automotive, electrical, and industrial construction industries due to their unique mechanical and chemical properties [4,5,6,7]. Their applications include radiator and heater hoses, window and door seals, O-rings and gaskets, accumulator bladders, wire and cable connectors and insulators, diaphragms, and weather stripping. Other major applications include roofing and waterproofing, such as bitumen modifications, facade and parapet sealants, expansion joints, and liners for pools and tanks. The typical working temperature range is relatively wide, from −45 °C to +150 °C, because the glass transition occurs at low temperatures. Moreover, the reinforcement of elastomers by adding the CB or silica fillers upgrades physical properties, such as the tear strength, tensile strength, hardness, abrasion resistance, and thermal properties [8,9,10,11,12,13,14]. 

Studies have been conducted on how fillers influence the permeability of polymer materials. The comparable effect of these fillers in reducing the permeability and prolonging the diffusion path for polymers has been investigated theoretically and experimentally [15,16,17,18,19,20,21]. The permeability of molecules through a polymer filled with well-dispersed and distributed plate-like particles was analyzed theoretically by Nielsen [22].

In addition, the potential of inorganic fillers with different shapes and sizes to reduce permeability was systematically approached by employing hydrogenated nitrile butadiene rubbers (HNBRs). The corresponding fillers, such as CB (N550) and precipitated silica, were incorporated at loadings up to 30 phr (parts per 100 parts of rubber). In the presence of 30 phr CB and precipitated silica, the diffusion coefficient was observed to decrease by 14–15% for both types of filler. Regarding the permeation coefficient, the incorporation of silica into HNBR has been shown to cause a reduction of 20%, whereas CB does not greatly affect the permeation rate of the composite [23]. The effects of CB filler on gas permeation in EPDM elastomers were investigated by employing a volumetric method. The observed effect of nonporous filler on the diffusivity and permeability was compared with the Maxwell theory prediction. This research was also conducted to clarify the correlation between transport properties and both the critical temperature and critical volume of the probing gas [24].

Under these research circumstances, it is necessary to investigate the effects of fillers on these related properties in rubber composites with the aid of an appropriate permeation measuring technique. Recently, we developed an effective volumetric analysis measurement method of emitted H_2_ gas using a graduated cylinder and diffusion analysis program [25,26]. The volumetric measurement is a very simple, inexpensive, and stable method to determine the permeation properties, regardless of specimen shape/dimension and gas species. Thus, the technique can be extended to the permeability measurement of various gases (He, N_2_, Ar) for specimen. In addition, the diffusion analysis program for simulating hydrogen transport property was upgraded for the use of various gases, different shapes (cylinder, sphere, and sheet) specimen at both modes of emission, and the remaining contents of gas. For the verification of the developed technique, we have already verified the volumetric analysis technique (VAT) in previous research [25] by comparing the results obtained by VAT with those by different methods, such as gas chromatography by thermal desorption analysis and gravimetric measurement by electronic balance for same samples. The results were found to be consistent with each other within uncertainty. 

Using the effective VAT, our investigations are focused on studies of the hydrogen permeation properties of blended EPDM mixed with CB and silica fillers. The effect of filler loading on hydrogen permeation is studied for these composites in an attempt to understand the corresponding sorption kinetics and diffusion dynamics. The current work also presented precise data on the gas permeability characteristics of polymer-blended materials. The hydrogen uptake, solubility, diffusivity, and permeability of the EPDM polymer composites blended with three kinds of fillers are systematically investigated as a function of the exposed pressure, filler content, and crosslink density. The final goal of this work is to find the appropriate EPDM composition for H_2_ gas sealing under high pressure and the correlation between the related physical properties.

## 2. Experimental

### 2.1. Sample Preparation and Composition

Ethylene propylene diene rubber (EPDM, Dow Chemical Company, Nodel^®^ IP 4760P, Midland, TX, USA) consisting of 65 wt% ethylene and 5.0 wt% ethylidene norbornene (ENB) was used as the main component for neat EPDM rubber. The compound recipe for the chemical composition for EPDM specimens with CB and silica fillers is given in Table 1 and Table 2, respectively, which includes one neat EPDM without any added filler, six samples with CB and three samples with silica filler. In this study, we employed two types of CB prepared using a high abrasion furnace (HAF, N330) and a semireinforcing furnace (SRF, N774) from Orion Engineer Carbon, which have particle sizes of 28–36 nm and 65 nm, respectively. The specific surface areas of the HAF and SRF are 76 m^2^/g and 30 m^2^/g, respectively. The commercial precipitated silica (Zeosil^®^ 175 MP) with a specific surface area of 175 m^2^/g was supplied by Solvay (Brussel, Belgium). The vulcanizates were filled with 20 phr, 40 phr, and 60 phr filler. For simplicity, the EPDM blends mixed with fillers were named EPDM HAFx, EPDM SRFy, and EPDM Sz, where x, y, and z indicate the phr content for HAF, SRF, and silica, respectively. For example, EPDM HAF40 is EPDM filled with HAF CB at 40 phr.

Two-stage mixing was employed using an internal mixer with two Banbury rotors and two open roll mills (model: PK-RM20140930, producer: Pungkwang CO., Hwaseong, Korea) of eight inches to prepare EPDM composites. The first stage of mixing (masterbatch) included compounding EPDM rubber, reinforcing fillers, such as carbon black and precipitated silica, and processing aids, such as ZnO and stearic acid, with an internal mixer (3 L kneader, Moriyama Co., Tokyo, Japan). The filling factor was fixed to 0.8, and the starting operation temperature of the kneader was set to 80 ℃. The rotor speed was set to 30 rpm. EPDM rubber was added to a 3 L kneader and kneaded for 3 min. Then, the reinforcing filler and the processing aids were incorporated for 10 min. In the second stage of mixing, open roll mills were used to add the curing agents and accelerating agent into the masterbatch composite. The mixer was set to a nip opening of 3 mm between the rolls. The masterbatch was added to the roller and mixed for 1 min. Sulfur, TBBS, and MBT were then added and mixed into a batch, which took approximately 2 min. The mixer nip was opened, and then, the finished batch was cut into sheets. The mixing time was kept uniform for all composites. Vulcanizate sheets of the composites with a thickness of 3 mm for measurement were prepared by compression molding in a hydraulic press at 160 ℃ based on the optimum cure time obtained from an oscillating disk rheometer.

### 2.2. Hydrogen Exposure Conditions

High-pressure chamber and purge conditions have been described in the preceding literature [25,26]. The specimen was exposed to hydrogen gas for 24 h in a pressure range from 1.2 MPa to 9.0 MPa. Hydrogen gas charging for 24 h is sufficient to attain an equilibrium state for gas sorption because of the fast diffusion rate of EPDM-based composites. After exposure to H_2_, the valve was opened, and the H_2_ in the chamber was released. After decompression, the elapsed time was recorded from the moment (*t* = 0) at which the high-pressure gas in the chamber was reduced to atmospheric pressure when the time was set to zero. As the specimen was loaded in the graduated cylinder after decompression, it took approximately 190 s~370 s to start the measurement. The gas content emitted during the inevitable time lag could be measured by offset determination via a diffusion analysis program.

## 3. Measurement Method and Data Analysis Program

### 3.1. Volumetric Analysis of Hydrogen

**Figure 1:**
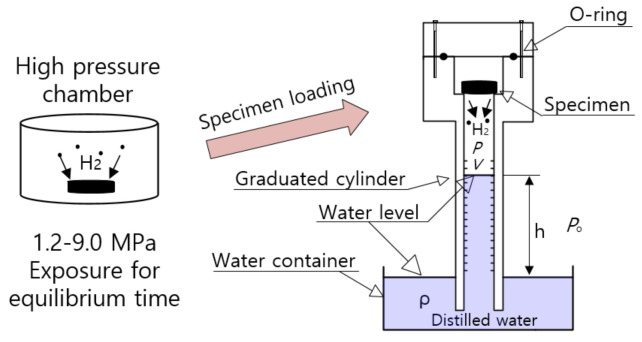
Configuration of the volumetric analysis system using a graduated cylinder after high-pressure exposure for 24 h and subsequent decompression.

Figure 1 shows the volumetric analysis system with a graduated cylinder to measure the released hydrogen gas. After exposure to the high-pressure chamber for 24 h and subsequent decompression, the specimen was loaded into the gas space of the top side in a graduated cylinder.

The hydrogen gas emitted from the specimen after decompression lowers the water level of the graduated cylinder. By reading the graduations on a standing graduated cylinder immersed partially in a water container, we measured the hydrogen gas amount released from the specimen. The gas inside the cylinder is governed by the ideal gas equation, *PV* = *nRT*, and R is the gas constant of 8.20544 × 10^−5^ m^3^·atm/(mol·K). *P* and *T* are the pressure and temperature, respectively, of the gas inside the cylinder with gas volume (*V*). The volume occupied by specimen is not included in *V*.

Thus, the increased number of moles (Δn) of emitted gas in the cylinder after decompression is obtained by measuring the increase in volume (ΔV) in the graduated cylinder, i.e., lowering of the water level, as follows [25,26]:

(1) $$\Delta n=\frac{(P_{o}-\rho gh)\Delta V}{RT}$$

where $P_{o}$ is the outside atmospheric pressure of the cylinder, ρ is the density of distilled water in the water container, and *g* is gravity. h is the height of the water level inside the graduated cylinder measured from the water levels in the water container.

The increased number of moles in the cylinder is converted to the mass concentration [$C\left(t\right)]$ of gas emitted from the specimen, as follows:

(2) $$C\left(t\right)\left[\mathrm{wt}\cdot \mathrm{ppm}\right]=\Delta n\left[\mathrm{mol}\right]\times \frac{m_{H2}\;\left[\frac{g}{\mathrm{mol}}\right]}{m_{sample}\left[g\right]}\times 10^{6}$$

where $m_{H2}$[g/mol] is the molar mass of H_2_ gas, 2.016 g/mol. $m_{sample}$ is the mass of the specimen. By measuring the change in the water level, the mass concentration of hydrogen emitted from rubber is acquired by Equation (2). A description of the volumetric method was also found in previous studies [25,26].

### 3.2. Diffusion Analysis Program

Assuming that the adsorption and desorption of gas is a diffusion-controlled process, the emitted gas content $C_{E}\left(t\right)$ in the desorption process is written as follows [27,28]:

(3) $$C_{E}\left(t\right)/C_{\infty }=1-\frac{32}{\pi^{2}}\times \left[\overset{\infty }{\underset{n=0}{\sum }}\frac{exp\left\{\frac{-{\left(2n+1\right)}^{2}\pi^{2}Dt}{l^{2}}\right\}}{{\left(2n+1\right)}^{2}}\right]\times \left[\overset{\infty }{\underset{n=1}{\sum }}\frac{exp\left\{-\frac{D\beta_{n}^{2}t}{\rho^{2}}\right\}}{\beta_{n}^{2}}\right]$$

Equation (3) is the solution to Fick’s second diffusion law for a cylindrical specimen under the boundary condition with an initially constant uniform gas concentration and constant concentration at the cylindrical surface. $C_{\infty }$ is the saturated hydrogen mass content at an infinitely long time, i.e., the total emitted mass concentration or hydrogen uptake in the desorption process. *D* is the diffusion coefficient of desorption. l and ρ is the thickness and radius, respectively, of cylindrical shaped specimen. $\beta_{n}$ is the root of the zero-order Bessel function.

**Figure 2:**
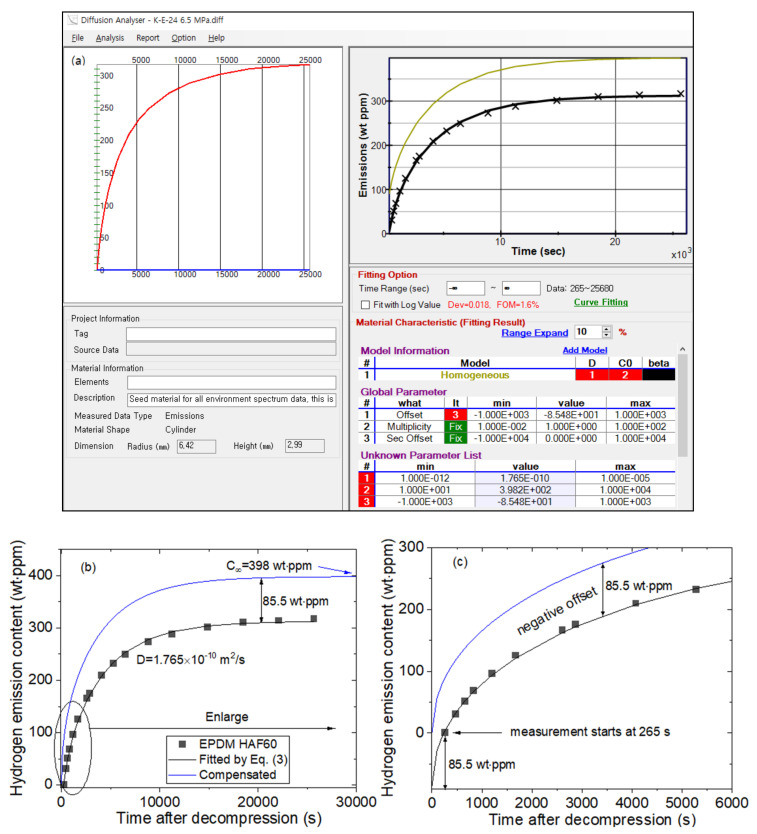
(**a**) Application example of the diffusion analysis program to obtain the hydrogen emission content and diffusion coefficient for EPDM HAF60 exposed to 6.5 MPa H_2_ for 24 h; (**b**) redrawn diffusion analysis results; and (**c**) enlargement of the elliptical part in (**b**).

To analyze the mass concentration data using Equation (3), a diffusion analysis program developed to calculate *D* and $C_{\infty }$, based on least-squares regression and a Nelder–Mead simplex optimization algorithm [25,26,29], is necessary. Figure 2 depicts a representative analysis result of the diffusion analysis program for EPDM H60 rubber exposed to 6.5 MPa H_2_. In the bottom left of Figure 2a, the radius and height (thickness) of the specimen with a cylindrical shape are the input. As shown in the right frame of Figure 2a, the × symbol and black line indicate the experimental data and line fitted with Equation (3), respectively. *D* and $C_{\infty }$ are obtained by substituting the hydrogen emission content at each time into Equation (3) and optimizing each parameter by the least squares method. The values *D* = 1.765 × 10^−10^ m^2^/s and $C_{\infty }$ = 398 wt·ppm with a negative offset of −85.5 wt·ppm (yellow line) are acquired, as shown in the unknown parameter list of Figure 2a. Dev = 0.018 indicates a standard deviation of 1.8% between the experimental data and the line fitted by Equation (3).

Notably, the hydrogen emission content is missing during the time lag between decompression and the start of measurement. Thus, the missed content is restored in the following manner. Figure 2b presents the redrawn results of Figure 2a. Figure 2c depicts an enlargement of the ellipse part in (b). Because the measurement started at 265 s after decompression due to the time lag, the emission value at t = 265 s is 0. However, the emitted hydrogen quantity should be 0 when t = 0. Therefore, we can compensate for the missing value between t = 0 and t = 265 s by upshifting (blue line) with an offset of 85.5 wt·ppm corresponding to a negative y value at t = 0 on the fitted black line in Figure 2c. The value indicated as the difference between the blue line and black line in Figure 2c is obtained by extrapolating the fitted black line satisfying the data according Equation (3) with the analysis program. The offset value is not neglected compared with $C_{\infty }$. Thus, the ultimate hydrogen emission content including the offset value is $C_{\infty }$ = 398 wt·ppm.

## 4. Results and Discussions

### 4.1. Filler Eeffect on Pressure-Dependent H_2_ Solubility

**Figure 3:**
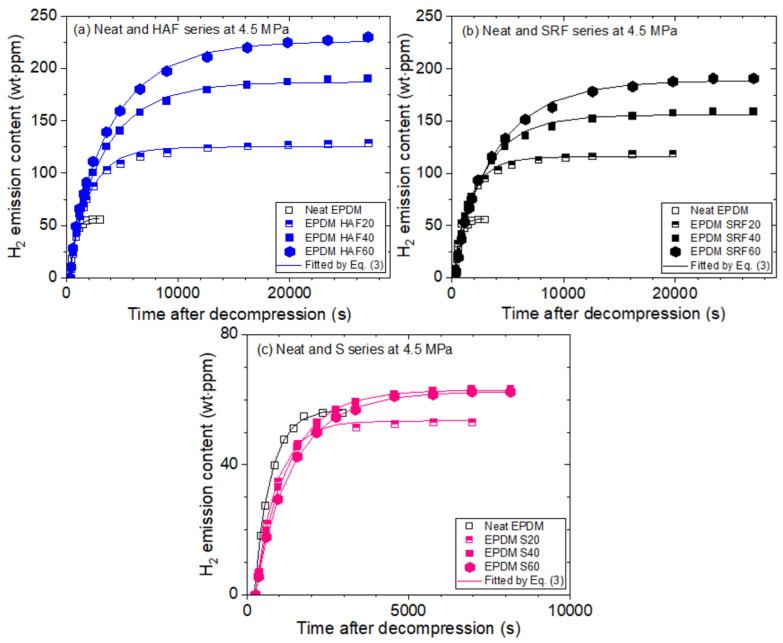
Time-varying H_2_ emission content of the (**a**) EPDM HAF series, (**b**) EPDM SRF series and (**c**) EPDM S series after exposure to 4.5 MPa H_2_ for 24 h and decompression. The solid lines represent the fitted results by Equation (3).

We measured the hydrogen emission content versus the elapsed time after decompression in the pressure range from 1.2 MPa to 9 MPa for neat EPDM and nine cylindrical EPDM composites filled with CB and silica. Figure 3 shows the representative H_2_ emission content versus time for the ten EPDM composites after exposure to 4.5 MPa H_2_ for 24 h. Figure 3a depicts the time-varying H_2_ emission content for neat EPDM and three EPDM composites with HAF CB fillers at 20 phr (EPDM HAF 20), 40 phr (EPDM HAF 40), and 60 phr (EPDM HAF 60). The hydrogen uptake increases and the diffusion rate decreases with increasing HAF filler content. Although a small difference in the filler effect exists, the filler effect on EPDM composites with SRF CB in Figure 3b displays a trend similar to that in Figure 3a. The small difference on filler influences is attributed to the different particle sizes and specific surface areas of the two kinds of fillers.

Moreover, as shown in Figure 3c, the variation in H_2_ emission content with the silica filler content does not show an appreciable change compared with that in neat EPDM, which is discussed in the following section.

**Figure 4:**
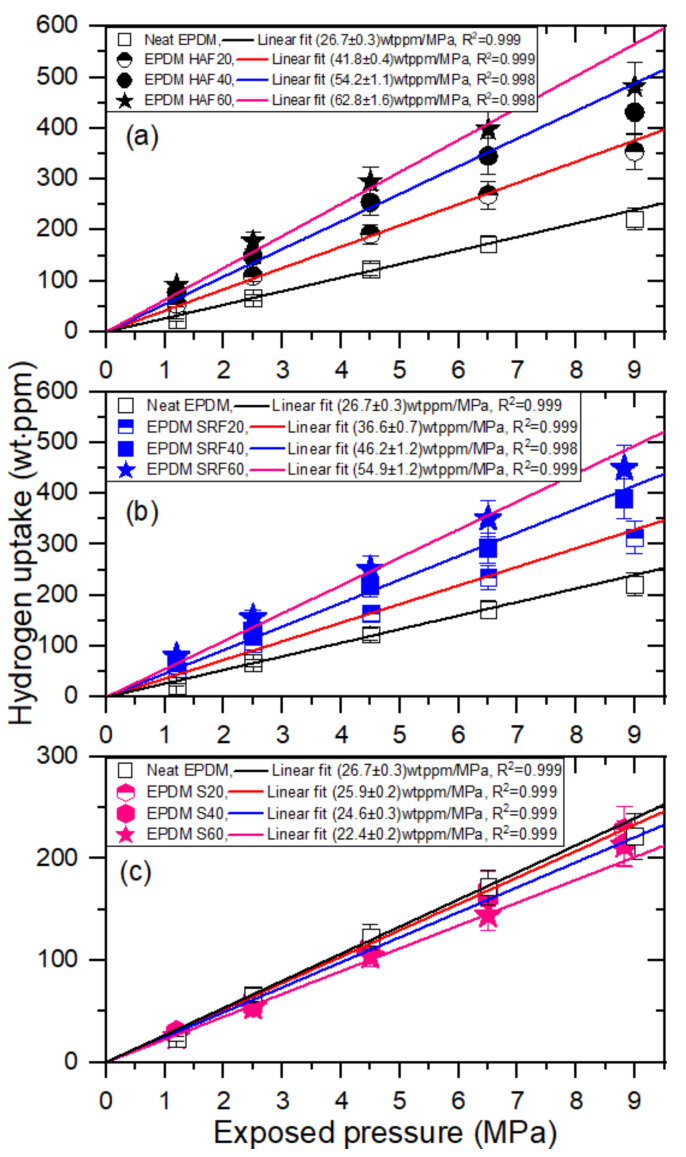
Hydrogen uptake ($C_{\infty }$) versus exposed pressure for the (**a**) EPDM HAF series, (**b**) EPDM SRF series, and (**c**) EPDM silica series. The lines indicated, as the slope of hydrogen uptake with respect to pressure, with a squared correlation coefficient R^2^, are the result of Henry’s law fit.

Figure 4 illustrates the hydrogen uptake as a function of pressure for the EPDM composites. Figure 4a–c show the pressure dependence of the hydrogen uptake EPDM composites blended with HAF CB filler, SRF CB filler, and silica filler, respectively. 

The hydrogen uptake for neat EPDM and nine filled EPDMs, as shown in Figure 4a–c, is proportional to the pressure up to 9 MPa H_2_ satisfying Henry’s law [30], which is the hydrogen uptake slope with regard to the pressure indicated by the slant lines. This finding implies that hydrogen does not dissociate and penetrates into the polymer as hydrogen molecules. According to Henry’s law, the hydrogen solubility (*S*) is acquired from the *C*_∞_ slope, with respect to pressure by the following relation:

(4) $$S\left[\frac{\mathrm{mol}}{m^{3}\cdot \mathrm{MPa}}\right]=\frac{C_{\infty }\;\mathrm{slope}\;\left[\frac{\mathrm{wt}\cdot \mathrm{ppm}}{\mathrm{MPa}}\right]\times 10^{6}\times d\left[\frac{g}{m^{3}}\right]}{m_{H_{2}}\left[\frac{g}{\mathrm{mol}}\right]}$$

where *m*_H2_ is the molar mass of hydrogen, *m*_H2_(g/mol) = 2.016 g/mol, and *d* is the density of the EPDM composites. According to Equation (4), the solubility of hydrogen for the EPDM composites is acquired.

**Figure 5:**
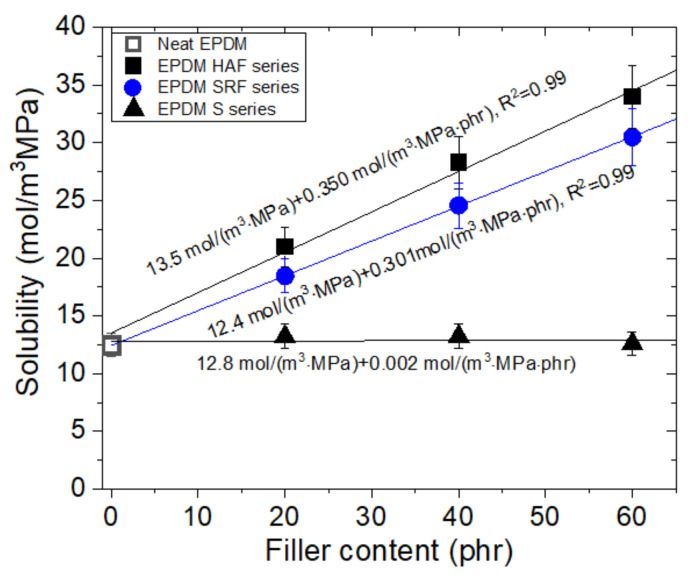
Variation in solubility versus different filler contents for filled EPDM composites. The three slanted lines are linear slopes of hydrogen solubility relative to the filler content.

Figure 5 depicts the linear variation in solubility versus filler content obtained in blended EPDM composites with filler, including neat EPDM. The solubility of HAF CB-filled EPDM increases linearly with increasing HAF CB filler content. From the slope with respect to the filler content, the hydrogen solubility per filler content for HAF CB-filled EPDM is 0.350 mol/(m^3^·MPa·phr), with an intersection of 13.5 mol/m^3^·MPa. Similar to that of HAF CB-filled EPDM, the solubility of SRF CB-filled EPDM composites increases linearly with the filler content. The hydrogen solubility per filler content of SRF CB-filled EPDM is found to be 0.301 mol/(m^3^·MPa·phr), with an intersection of 12.4 mol/m^3^·MPa. However, that of the silica-filled EPDM barely increases with increasing filler content. The solubility of silica-filled EPDM is 0.002 mol/(m^3^·MPa·phr), with an intersection of 12.8 mol/m^3^·MPa, implying that silica in silica-filled EPDM composites hardly promotes the adsorption of hydrogen.

From experimental investigations of the solubility slope, we discovered that the contribution of hydrogen solubility comprises two contributions: from absorbed hydrogen in the parent polymer network corresponding to an intersection value and from adsorbed hydrogen at the filler surface corresponding to the slope. Thus, the solubility value for the filler in the filled EPDM composites is predicted if the filler content is given. The small difference in slope in the HAF and SRF CB filler effects may be related to the particle size and specific surface area of the filler. EPDM composites with HAF CB filler with a smaller filler size and larger specific surface area than those for SRF CB filler effectively contribute to the increase in solubility. Moreover, hydrogen adsorbed by CB accumulates at the boundary interface of CB particles [31,32]. As the size of CB is smaller, the specific surface area increases, and thus, a large amount of hydrogen exists around the boundary of CB. The results of previous studies [31,32] are satisfactorily consistent with our experimental findings.

Regarding the larger solubility in CB-filled EPDM than in silica-filled EPDM, hydrogen molecules were adsorbed at the interface between CB and the matrix in a rubber structure, unlike the case with silica filler. Furthermore, hydrogen trapping by CB was confirmed to be one of the reasons for the increased hydrogen content [31]. Consequently, as expected, the solubility of CB-filled EPDM was found to be increased compared with that of unfilled and silica-filled EPDM. The solubility values of the silica-filled EPDM were nearly equal to that of neat EPDM, although the specific surface area of silica was larger than that of CB. This result indicates that CB and silica differ in terms of the interfacial structure. Therefore, it is inferred that the hydrogen solubility of filled rubbers is influenced not only by the surface area of fillers but also by the interface structure between the filler and polymer matrix.

### 4.2. Filler Effect on H_2_ Diffusivity

**Figure 6:**
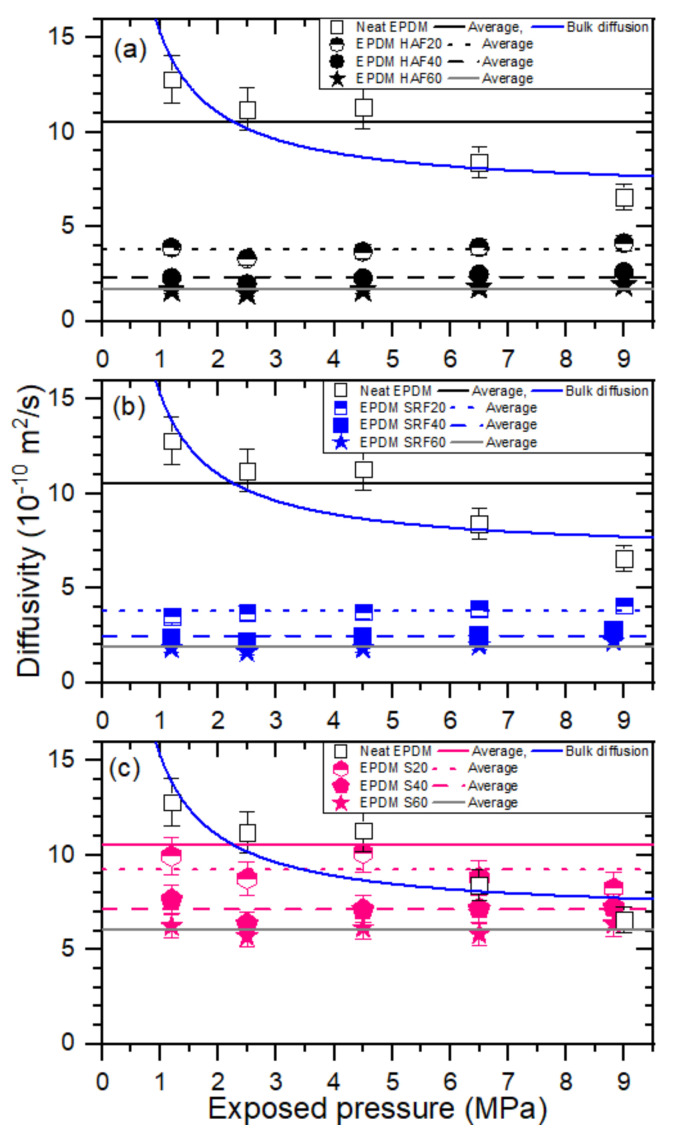
Hydrogen diffusivity (*D*) versus exposed pressure for the (**a**) EPDM HAF series, (**b**) EPDM SRF series and (**c**) EPDM S series. The blue line for neat EPDM represents the result of bulk diffusion fitting. The horizontal lines for neat EPDM and blended EPDMs containing filler represent the average diffusivity. The same scale on the x-axis and y-axis is presented for comparison.

The hydrogen diffusivity in neat EPDM and the filled EPDM composites as a function of pressure is presented in Figure 6a–c. Neat EPDM exhibits only pressure-dependent diffusion behavior, which is attributed to bulk diffusion behavior. This aspect has been observed and analyzed by fractal theory in other studies [28,33]. The bulk diffusion coefficient for neat EPDM is inversely proportional to pressure and is associated with the mean free path between H_2_ molecules. Bulk diffusion is predominant if the mean free path (λ) is less than the pore diameter found in large pores or high-pressure gas diffusion. The bulk diffusion coefficient ($D_{B})$ can be expressed as follows [34]:

(5) $$D_{B}=\frac{1}{3}\lambda \upsilon =\frac{1}{3}\frac{5}{8}\frac{\mu }{P}\sqrt{\frac{RT\pi }{2M}}\upsilon$$

where μ is the viscosity of the diffusion molecule in units of kg m/s, P is the pressure, υ is the average gas molecular velocity, R is the gas constant, T is the temperature, and M is the gas molar mass. The factor 5/8 considers the Maxwell–Boltzmann distribution of molecular velocity. The experimental results of the diffusion coefficient in Figure 6 for neat EPDM are fitted by Equation (5), as indicated by the blue line. The decrease in the bulk diffusion coefficient is attributed to a decrease in the mean free path with increasing pressure.

On the other hand, the diffusivity of EPDM composites blended with fillers in Figure 6a–c does not exhibit pressure dependence. Thus, we take the average diffusivity as a representative value. No pressure dependence on diffusivity is attributed to a prior decrease in the mean free path of hydrogen molecules or an increase in tortuosity by introducing a hydrogen-impermeable filler into the polymer network. 

It is important to note that the norbornene macromolecular groups in EPDM restrict the chains from being closer to each other. Thus, H_2_ molecules are available to occupy more empty space, leading to a diffusivity on the order of 10^−10^ m^2^/s, which is larger than that of other polymers, such as nitrile butadiene rubber (NBR) and fluoroelastomers [25,26].

**Figure 7:**
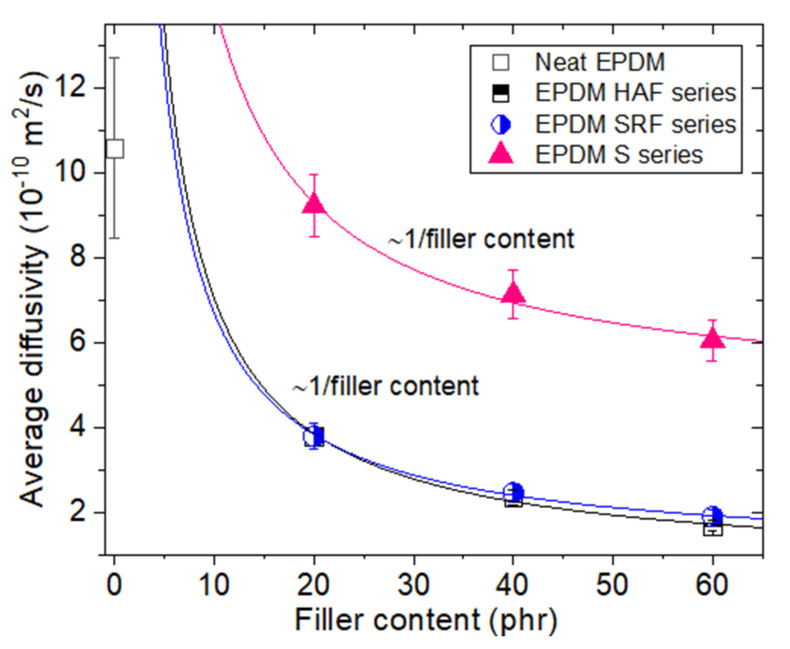
Average diffusivity versus filler content for neat EPDM and EPDM composites blended with filler.

Figure 7 shows the variation in average diffusivity for neat EPDM and blended EPDM composites. All fillers suppress hydrogen diffusion due to the increased tortuosity. The similar filler effect is found in other literature [35,36,37]. As a result, the average diffusivity of filled EPDM composites decreases with increasing filler content (~1/filler content), irrespective of the type of filler. The average diffusivity for all specimens converges on a fixed value at a maximum filler content above 60 phr. The larger decrease in the diffusivity for CB-filled EPDM than for silica-filled EPDM may be related to the attractive trapping of adsorbed hydrogen by the CB filler, thus requiring a large activation energy compared to that for the silica filler. According to previous investigations of NBR blended with fillers, the activation energy, that is, potential barriers for hydrogen diffusion in HAF CB-filled NBR, was found to be larger than that in both neat NBR and silica-filled NBR. The similar filler effect is observed in other literature [36,38,39]. The difference in the effect on diffusivity between the two fillers observed in the EPDM composites could be interpreted in terms of activation energy, similar to that in NBR.

### 4.3. Correlation between the Permeation Parameters and Crosslink Density

**Figure 8:**
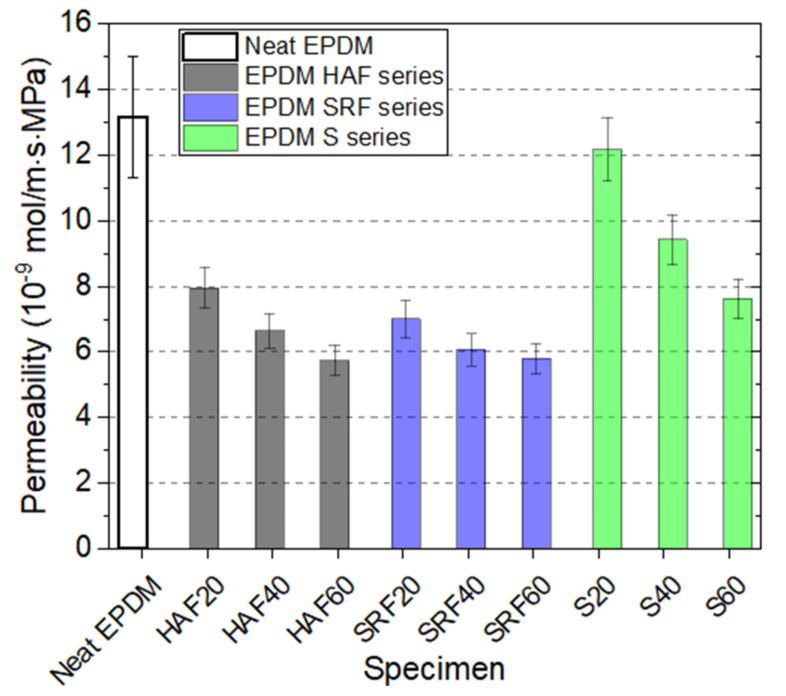
Permeability of neat EPDM and filled EPDM composites.

The permeability (P) was obtained by multiplying the solubility (S) and average diffusivity. Figure 8 demonstrates the permeability bar for neat EPDM and the filled EPDM composites. The bar indicates the expanded uncertainty estimated in the previous study. The permeability of EPDM composites decreases with increasing filler content, irrespective of the filler species. The filler influence on permeability is similar to that on diffusivity, as shown in Figure 7. The filler effects on permeability are more powerful in EPDM composites with HAF CB and SRF CB fillers than with silica fillers. In particular, EPDM HAF60 and EPDM SRF 60 with the lowest hydrogen permeability could be appropriate sealing candidates for high-pressure gas sealing, although numerous factors must be considered.

We also present the permeation characteristics of the EPDM determined in this work in Table 3, which is merely a database showing the results of solubility, average diffusivity, and permeability, which are already discussed in Figure 5, Figure 6, and Figure 8, respectively. 

We reconsidered on the impact of the amount of fillers, that is, the variation of solubility, diffusivity, and permeability versus the corresponding filler content in filled-EPDM composites. The solubility in Figure 5 increases smoothly with increasing the all filler content used. Meanwhile, even with small CB filler content of 20 phr, the diffusivity (Figure 7) and permeability (Figure 8) in CB filled-EPDM composites abruptly decreases, unlikely those in silica filled-EPDM with smooth change. This implies that small CB filler content can effectively control the permeation parameters.

The crosslink density was determined by equilibrium swelling. The swelling experiments were conducted by immersing the sample in terahydrofuran (THF) at ambient temperature for 72 h. The weight of rubber sample before and after the immersion was measured. The crosslink density (ν) of units in (mol/g) for the EPDM polymer composites is calculated according to the Flory-Rehner equations, Equations (6) and (7) [40,41,42]:

(6) $$\nu =\frac{1}{2M_{c}}=-\frac{\ln\left(1-V_{1}\right)+V_{1}+\chi V_{1}^{2}\;}{2\rho_{r}V_{0}\left(V_{1}^{\frac{1}{3}}-\frac{V_{1}}{2}\right)}$$

(7) $$V_{1}=\frac{\frac{W_{d}-W_{f}}{\rho_{r}}}{\frac{W_{d}-W_{f}}{\rho_{r}}+\frac{W_{s}-W_{d}}{\rho_{s}}}$$

where$\;M_{c}$ is the average molecular weight between crosslinks, $V_{0}$ is the molar volume of the solvent (cm³/mol) and $V_{1}$ is the volume fraction of rubber in the swollen network at equilibrium. $W_{d}$ is the weight of the unswollen sample, $W_{f}$ is the weight of the filler in the compound, $W_{s}\;$is the weight of the swollen sample, $\rho_{r}\;$is the density of EPDM composites, $\rho_{s}\;$is the density of the THF, and χ is the polymer–solvent interaction parameter (χ = 0.501). 

**Figure 9:**
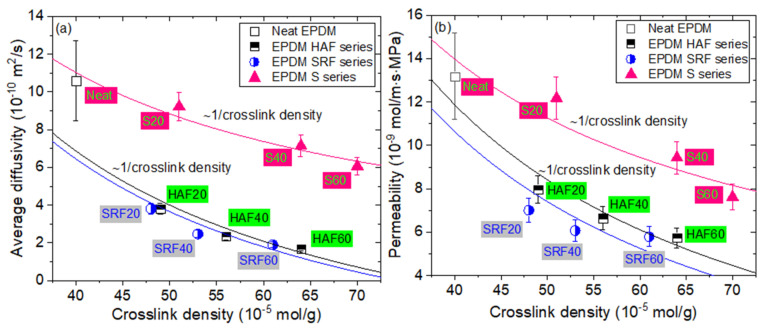
Linear correlation between the (**a**) average diffusivity and (**b**) the permeability and reciprocal crosslink density for neat EPDM and EPDM composites blended with filler. The solid lines represent the least squares fitting results, indicated as the average diffusivity and permeability proportional to ~1/crosslink density.

To clarify the origin of the permeation phenomenon, we tried to find the correlation between permeation parameters and crosslink density of the EPDM composites. Figure 9 displays the variation in both the average diffusivity and permeability versus the crosslink density of the EPDM specimen. With increasing crosslink density, as expected, both the diffusivity and permeability decrease. The average diffusivity (or permeability) proportional to the reciprocal crosslink density is observed for all composites, irrespective of the filler species. Similar to filler-dependent solubility and diffusivity, as shown in Figure 5 and Figure 7, respectively, the CB filler is more effective than the silica filler. This finding implies that the crosslink density of the specimen could also be a factor controlling diffusion and permeation.

## 5. Conclusions

An effective volumetric analysis technique is applied successfully to characterize hydrogen permeation with a diffusion analysis program by employing the Nelder–Mead simplex optimization algorithm. Filler effects on H_2_ pressure-dependent permeation properties for neat EPDM and EPDM blended with CB and silica fillers are systematically investigated in the pressure range of 1.2 MPa~9 MPa. This technique determines the total uptake, solubility, diffusivity and permeability of hydrogen for ten EPDM composites as a function of the exposure pressure, filler species, and filler content.

The filler effects for all EPDM composites display particular characteristics depending on the filler species and content. For the pressures investigated in this work, hydrogen uptake for all specimens follows Henry’s law up to 9 MPa. This result indicates that hydrogen does not dissociate and penetrates into the polymer as a hydrogen molecule. The hydrogen solubility linearly increases with increasing filler concentration. Hydrogen solubility consists of two contributions from hydrogen absorption in the parent polymer network and hydrogen adsorption at the corresponding CB filler surface. The hydrogen solubility of the parent polymer is 13 ± 5 mol/(m^3^·MPa). The hydrogen solubility per filler is determined to be 0.350 mol/(m^3^·MPa·phr) for the HAF CB filler and 0.301 mol/(m^3^·MPa·phr) for the SRF CB filler, while the silica filler exhibits barely any hydrogen solubility. Neat EPDM demonstrates bulk diffusion behavior related to a decrease in the mean free path with increasing pressure. As the filler content increases, the pressure dependence on diffusivity weakens. The filler effect on hydrogen solubility and diffusion is observed in the order of HAF CB filler~SRF CB filler > silica filler, which is related to the specific surface area of the CB particles. The larger solubility/diffusivity in CB filler than in silica filler originates from adsorbed trapping, the interfacial structure at the boundary of the polymer/filler, and different activation energies of diffusion motion.

An inverse correlation between the average diffusivity or the permeability and crosslink density is found for CB-filled and silica-filled EPDM. The permeation effect in terms of crosslink density is similar to that of the permeability parameter versus filler content. EPDM HAF60 and EPDM SRF60 with the lowest hydrogen permeability could be appropriate candidates for high-pressure hydrogen gas sealing.

## Figures and Tables

**Table 1:** Composition of the EPDM series with HAF and SRF CB fillers.

Chemical Composition	Neat EPDM	EPDM HAF20	EPDM HAF40	EPDM HAF60	EPDM SRF20	EPDM SRF40	EPDM SRF60
EPDM	100	100	100	100	100	100	100
ZnO	3.0	3.0	3.0	3.0	3.0	3.0	3.0
St/A	1.0	1.0	1.0	1.0	1.0	1.0	1.0
HAF N330	-	20	40	60	-	-	-
SRF N774	-	-	-	-	20	40	60
S	1.5	1.5	1.5	1.5	1.5	1.5	1.5
TBBS	1.0	1.0	1.0	1.0	1.0	1.0	1.0
MBT	0.5	0.5	0.5	0.5	0.5	0.5	0.5

St/A: stearic acid, TBBS: N-tert-butyl-2-benzothiazole sulfenamide, MBT: 2-mercaptobenzothiazole.

**Table 2:** Composition of the EPDM series with silica fillers.

Chemical Composition	EPDM S20	EPDM S40	EPDM S60
EPDM	100	100	100
ZnO	3.0	3.0	3.0
St/A	1.0	1.0	1.0
Silica S-175	20	40	60
Si-69	1.6	3.2	4.8
PEG	0.8	1.6	2.4
S	1.5	1.5	1.5
TBBS	1.0	1.0	1.0
MBT	0.5	0.5	0.5

Si-69: silane coupling agent, PEG: polyethylene glycol.

**Table 3:** Summary of H_2_ permeation parameters for EPDM composites.

Specimen	Solubility [mol/m^3^·MPa]	Average Diffusivity [10^−10^ m^2^/s]	Permeability [10^−9^ mol/m·s·MPa]
Neat EPDM	12.4 ± 1.0	10.6 ± 2.1	13.2 ± 2.6
EPDM HAF20	20.9 ± 1.7	3.80 ± 0.3	7.96 ± 0.64
EPDM HAF40	28.3 ± 2.3	2.35 ± 0.19	6.65 ± 0.53
EPDM HAF60	34.0 ± 2.7	1.69 ± 0.14	5.74 ± 0.46
EPDM SRF20	18.5 ± 1.5	3.80 ± 0.30	7.01 ± 0.56
EPDM SRF40	24.6 ± 2.0	2.47 ± 0.20	6.07 ± 0.49
EPDM SRF60	30.5 ± 2.5	1.90 ± 0.15	5.79 ± 0.46
EPDM S20	13.2 ± 1.1	9.23 ± 0.74	12.2 ± 1.0
EPDM S40	13.2 ± 1.1	7.14 ± 0.57	9.43 ± 0.75
EPDM S60	12.6 ± 1.0	6.06 ± 0.49	7.62 ± 0.61

## Data Availability

The data used to support the findings of this study are available from the corresponding author upon request.

## References

[1] Ravishankar P. (2012). Treatise on EPDM. Rubber Chem. Technol..

[2] Green M.M., Wittcoff H. (2003). Organic Chemistry Principles and Industrial Practice.

[3] Louie D.K., Louie D.K. (2005). Elastomers. Handbook of Sulphuric Acid Manufacturing.

[4] Delor-Jestin F., Lacoste J., Barrois-Oudin N., Cardinet C., Lemaire J. (2000). Photo-, thermal and natural ageing of ethylene–propylene–diene monomer (EPDM) rubber used in automotive applications. Influence of carbon black, crosslinking and stabilizing agents. Polym. Degrad. Stab..

[5] Kumar A., Choudhary R., Kumar A. (2020). Characterisation of asphalt binder modified with ethylene–propylene–diene–monomer (EPDM) rubber waste from automobile industry. Road Mater. Pavement Des..

[6] Lee S.H., Park S.Y., Chung K.H., Jang K.S. (2021). Phlogopite-reinforced natural rubber (NR)/ethylene-propylene-diene monomer rubber (EPDM) composites with aminosilane compatibilizer. Polymers.

[7] Surya I., Muniyadi M., Ismail H. (2021). A review on clay-reinforced ethylene propylene diene terpolymer composites. Polym. Compos..

[8] Nanda M., Chaudhary R.N.P., Tripathy D.K. (2010). Dielectric relaxation of conductive carbon black reinforced chlorosulfonated polyethylene vulcanizates. Polym. Compos..

[9] Das N.C., Chaki T.K., Khastgir D., Chakraborty A. (2002). Electrical and mechanical properties of conductive carbon black filled EVA, EPDM and their blends. Kautsch. Gummi Kunstst..

[10] Stockelhuber K.W., Svistkov A.S., Pelevin A.G., Heinrich G. (2011). Impact of filler surface modification on large scale mechanics of styrene butadiene/silica rubber composites. Macromolecules.

[11] Li W., Peng W., Ren S., He A. (2019). Synthesis and characterization of trans-1,4-poly(butadiene-co-isoprene) rubbers (TBIR) with different fraction and chain sequence distribution and its influence on the properties of natural rubber/TBIR/carbon black composites. Ind. Eng. Chem. Res..

[12] Roy C., Chaala A., Darmstadt H. (1999). The vacuum pyrolysis of used tires: End-uses for oil and carbon black products. J. Anal. Appl. Pyrolysis.

[13] Hashim A.S., Azahari B., Ikeda Y., Kohjiya S. (1998). The effect of bis(3-triethoxysilylpropyl) tetrasulfide on silica reinforcement of styrene-butadiene rubber. Rubber Chem. Technol..

[14] Brinke J.W.T., Debnath S.C., Reuvekamp L.A.E.M., Noordermeer J.W.M. (2003). Mechanistic aspects of the role of coupling agents in silica–rubber composites. Compos. Sci. Technol..

[15] Huabing L., Jiandong Z., Biqin D., Feng X. (2020). Effect of Lamellar Inorganic Fillers on the Properties of Epoxy Emulsion Cement Mortar. Int. J. Concr. Struct. Mater..

[16] Ahn W., Cornigli D., Varghese D., Nguyen L., Krishnan S., Reggiani S., Alam M.A. (2020). Effects of Filler Configuration and Moisture on Dissipation Factor and Critical Electric Field of Epoxy Composites for HV-ICs Encapsulation. IEEE Trans. Compon. Packag. Manufact. Technol..

[17] Beckmann W. (1991). Gasdurchla¨ssigkeit von elastomeren. Kautsch. Gummi Kunstst..

[18] Wolf C., Angellier-Coussy H., Gontard N., Doghieri F., Guillard V. (2018). How the shape of fillers affects the barrier properties of polymer/non-porous particles nanocomposites: A review. J. Membr. Sci..

[19] Rosca C., Giese U., Schuster R.H. (2006). Investigation of diffusion of phthalates in nitrile rubber by means of FT-IR-spectroscopy. Kautsch. Gummi Kunstst..

[20] Bastani D., Esmaeili N., Asadollahi M. (2013). Polymeric mixed matrix membranes containing zeolites as a filler for gas separation applications: A review. J. Ind. Eng. Chem..

[21] Andrady A.L., Merkel T.C., Toy L.G. (2004). Effect of particle size on gas permeability of filled superglassy polymers. Macromolecules.

[22] Nielsen L.E. (1967). Models for the permeability of filled polymer systems. J. Macromol. Sci. Chem..

[23] Yang X., Giese U., Schuster R.H. (2008). Characterization of permeability of elastomers-part I-HNBR. Kautsch. Gummi Kunstst..

[24] Rutherford S., Kurtz R., Smith M., Honnell K., Coons J. (2005). Measurement and correlation of sorption and transport properties of ethylene-propylene-diene monomer (EPDM) elastomers. J. Membr. Sci..

[25] Jung J.K., Kim I.G., Kim K.T., Ryu K.S., Chung K.S. (2021). Evaluation techniques of hydrogen permeation in sealing rubber materials. Polym. Test..

[26] Jung J.K., Kim I.G., Jeon S.K., Kim K.T., Baek U.B., Nahm S.H. (2021). Volumetric analysis technique for analyzing the transport properties of hydrogen gas in cylindrical-shaped rubbery polymers. Polym. Test..

[27] Crank J. (1975). The Mathematics of Diffusion.

[28] Yang Y., Liu S. (2019). Estimation and modeling of pressure-dependent gas diffusion coefficient for coal: A fractal theory-based approach. Fuel.

[29] Abdel-Basset M., Mohamed R., Mirjalili S. (2021). A Novel Whale Optimization Algorithm Integrated With Nelder-Mead Simplex for Multi-Objective Optimization Problems. Knowl.-Based Syst..

[30] Sander R. (2015). Compilation of Henry’s law constants (version 4.0) for water as solvent. Atmos. Chem. Phys..

[31] Yamabe J., Nishimura S. (2009). Influence of fillers on hydrogen penetration properties and blister fracture of rubber composites for O-ring exposed to high-pressure hydrogen gas. Int. J. Hydrog. Energy.

[32] Yamabe J., Nishimura S. (2011). Influence of carbon black on decompression failure and hydrogen permeation properties of filled ethylene-propylene–diene–methylene rubbers exposed to high-pressure hydrogen gas. J. Appl. Polym. Sci..

[33] Wang Y., Liu S. (2016). Estimation of pressure-dependent diffusive permeability of coal using methane diffusion coefficient: Laboratory measurements and modeling. Energy Fuels.

[34] Welty J.R., Wicks C.E., Wilson R.E. (1984). Fundamentals of Momentum, Heat, and Mass Transfer.

[35] Gabriele G.G., Ahmed I., Domenico B., Ahmad E.K. (2020). Composite Polymers Development and Application for Polymer Electrolyte Membrane Technologies—A Review. Molecules.

[36] Gabriele C., Paola B. (2022). A Review of the Recent Progress in the Development of Nanocomposites Based on Poly(ether-block-amide) Copolymers as Membranes for CO2 Separation. Polymers.

[37] Zhang Y., Liu Q., Zhang S., Zhang Y., Cheng H. (2015). Gas barrier properties and mechanism of kaolin/styrene–butadiene rubber nanocomposites. Appl. Clay Sci..

[38] Markus M.M., Frank F., Manfred K. (2014). Effect of Filler Surface Activity and Morphology on Mechanical and Dielectric Properties of NBR/Graphene Nanocomposites. Rubber Chem. Technol..

[39] Yang Z., Dietmar D. (2019). Influence of Filler Content and Filler Size on the Curing Kinetics of an Epoxy Resin. Polymers.

[40] Lee J.Y., Park N., Lim S., Ahn B., Kim W., Moon H., Paik H.J., Kim W. (2017). Influence of the silanes on the crosslink density and crosslink structure of silica-filled solution styrene butadiene rubber compounds. Compos. Interfaces.

[41] Marzocca A.J. (2007). Evaluation of the polymer–solvent interaction parameter for the system cured styrene butadiene rubber and toluene. Eur. Polym. J..

[42] Hrnjak-Murgic Z., Jelencic J., Bravar M., Marovic M. (1998). Influence of the network on the interaction parameter in system EPDM vulcanizate-solvent. J. Appl. Polym. Sci..

